# Postgraduate Surgical Training in the UK: the Trainees’ Perspective

**DOI:** 10.1007/s12262-021-03112-6

**Published:** 2021-09-12

**Authors:** Anna Rose, Noel Aruparayil

**Affiliations:** 1 Canniesburn Department of Plastic & Reconstructive Surgery, Glasgow Royal Infirmary, Glasgow, UK; 2 Association of Surgeons in Training, London, UK; 3 Global Anaesthesia Surgery and Obstetrics Collaboration, London, UK; 4 Barnsley District General Hospital, Barnsley, UK

**Keywords:** Surgical training, UK, Trainees, Education, Webinars, COVID-19, Pandemic, Virtual learning, Simulation

## Abstract

Over the last 20 years, surgical training in the United Kingdom (UK) has changed dramatically. There have been considerable efforts towards creating a programme that delivers the highest standard of training while maintaining patient safety. However, the journey to improve the quality of training has faced several hurdles and challenges. Recruitment processes, junior doctor contracts, flexible working hours and equality and diversity have all been under the spotlight in recent times. These issues, alongside the extended surgical team and the increasingly recognised importance of trainee wellbeing, mean that postgraduate surgical training is extremely topical. Alongside this, as technology has evolved, this has been incorporated into all aspects of training, from recruitment to simulated training opportunities and postgraduate examinations. The coronavirus (COVID-19) pandemic has brought technology and simulation to the forefront in an attempt to compensate for reduced operative exposure and experience, and has transformed the way that we learn and work. In this article, we reflect on the UK surgical trainee experience and discuss areas of success as well as highlighting potential areas for improvement going forward.

## Training: Past, Present, Future

Surgical training initially began as an apprentice-style model with no specific duration of or defined career progression [1]. The Calman training system reforms implemented in 1996 by Chief Medical Officer Sir Kenneth Calman introduced a focused training system with structured teaching, specified competencies and supervised learning as a cost-neutral initiative [2]. As a result of these changes, the overall time spent in training, both day to day and also in cumulative years, has steadily reduced, but this has also caused a perceived reduced flexibility of career choices for the so-called pluripotent surgical trainee [3]. These changes were focused on developing a specialist workforce leading on to ‘Specialist Registrar’ posts to replace the previous ‘Senior Registrar’. A maximum of seven years was required to progress as a consultant. However, no changes were made for the training needs of Senior House Officers (SHO) giving rise to a so-called lost tribe [4]. In 2002, two documents entitled ‘Unfinished Business’ and ‘Choice and Opportunity’ were published with the aim of improving training requirements and progression for SHO, Non-Consultant Career grade (NCCG) and overseas doctors [5]. In 2005, this directly led to the launch of the reform programme ‘Modernising Medical Careers’ (MMC) and ‘Medical Training Application System’ (MTAS), offering structured, yet flexible, training opportunities. However, the MMC programme seriously failed to establish a streamlined framework for speciality training and led to several corrective actions and, ultimately, the restructuring of speciality training [6]. ‘Core Surgical training’ and ‘Speciality training’ posts were then introduced with competitive selection processes in place. Trainees received a certificate for completion of training (CCT) within surgery and entered the specialist register held by the General Medical Council (GMC). Introduction of consultant-led service and the European Working Time Directive (EWTD) resulted in 48 h of working time for trainee doctors. The 2013 ‘Shape of Training’ review [7] further emphasised the need for a shift from service provision to structured training. In an attempt to rectify this, the latest ‘Improving Surgical Training’ (IST) pilot was designed by the Royal College of Surgeons and Health Education England (HEE) [8]. This re-introduced the concept of ‘run-through’ specialties. The pilot aimed to redress the balance between training and service provision, particularly focusing on more in-hours training, establishing cross-specialty and cross-professional competencies, improving the role of trainers by increasing their dedicated training time, investing in focused training opportunities including simulation strategies and expanding the surgical team. In March 2020, when elective operating was largely suspended due to the COVID-19 pandemic, trainees lost a significant amount of operating experience and this has further highlighted the importance of training ‘tomorrow’s surgeons’[9] The detailed framework for specialty training programmes has been agreed by the four UK health departments and has been published in the eighth edition of the Reference Guide for Postgraduate Foundation and Specialty Training in the UK (also known as the Gold Guide) [10].

## Recruitment Process

**Fig. 1:**
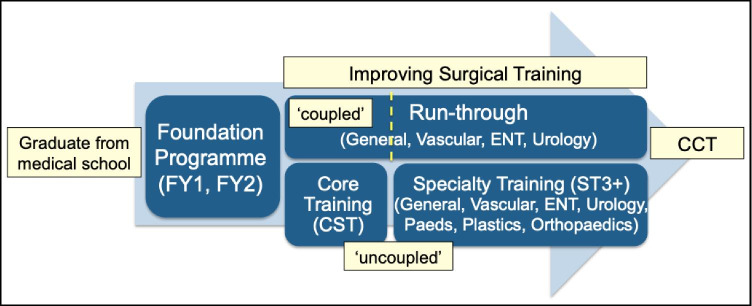
Flowchart demonstrating path of surgical training

As it stands, UK medical graduates enter into foundation year (FY) training for 2 years, upon completion of which they have full registration with the General Medical Council (GMC) and are eligible to apply for core surgical training (CST) at national selection to a programme either ‘coupled’ with a higher surgical training post (also known as ‘run-through’) or a standalone ‘uncoupled’ 2-year post. Some surgical specialties (namely Neurosurgery, Maxillofacial surgery, Cardiothoracic and Orthopaedics) can progress directly into specialty training (see Fig. 1). Prior to the COVID-19 pandemic, completion of both membership of the Royal College of Surgeons (MRCS) examinations, part A and B, was required in order to progress from CST to specialty training (ST) posts. Since then, for a limited period of time, trainees have been permitted to progress with MRCS part A only.

For trainees interested in a career in academic surgery, after graduating from medical school, trainees can apply for a 2-year Specialised Foundation Programme, previously known as Academic Foundation Programme. The programme focuses on research, leadership and management or medical education [11]. Trainees can progress as academic clinical fellows and lecturers through a competitive selection process.

In the UK, surgical education is managed regionally by Local Education and Training Boards. The Joint Committee on Surgical Training (JCST) is responsible for curriculum development and quality assurance of all the surgical training programmes in the ten defined surgical specialities via Specialty Advisory Committees: Cardiothoracic Surgery, General Surgery, Neurosurgery, Oral and Maxillofacial Surgery, Otolaryngology, Paediatric Surgery, Plastic Surgery, Trauma and Orthopaedics, Urology and Vascular Surgery. The end of training is marked by the award of a ‘Certificate of Completion of Training’ (CCT) which requires completion of the intercollegiate fellowship examinations, completion of surgical training competency-based assessments, demonstration of management and leadership skills and logbook evidence as outlined by the JCST. The trainees pay a tax-deductible annual fee of £260 to JCST to maintain an electronic portfolio that is monitored by clinical and educational supervisors.

## Role of the Royal Colleges

There are currently 4 Royal Colleges in the UK: The Royal College of Surgeons of England (RCS Eng), The Royal College of Surgeons of Edinburg (RCS Ed) and The Royal College of Physicians and Surgeons of Glasgow (RCPSG). The Royal Colleges offer a range of educational and training resources for both the UK and international trainees. Some colleges were established in the early 1500 s and have continued to provide learning and advancement in surgery. The membership or fellowship exams are a key step in training progression, awarding full membership status provides a title of ‘Miss/Ms/Mr’ for successful trainees. Royal Colleges play a crucial role in developing the surgical curriculum, lobbying for trainees and promoting and supporting surgical training.

Since the publication of the Lancet Commission on Global Surgery [12], all colleges have increased their international engagement by organising events and offering small bursaries. The Royal Colleges are also promoting and assisting international doctors to gain surgical experience in the UK. This can happen through the International Postgraduate Deanery (IPD) of RCS Ed over 12–24 months. RCS England similarly offers assistance to visiting specialists and refugee surgeons. International doctors can apply for a work permit through the Medical Training Initiatives (MTI) programme. International doctors can individually apply for training posts through a competitive process but NHS Employers cannot target countries with a lower workforce [13]. However, this does not limit individuals applying for employment in the UK.

## Role of Trainee Organisations

### UK

The Association of Surgeons in Training (ASiT) is an independent professional body that works to promote the highest standards in surgical training [14]. It was originally established in 1976 as a forum for senior registrars and now counts numerous professional leaders and Royal College Presidents amongst its alumni. ASiT is one of the largest surgical speciality associations in the UK with around 3500 members. As a formal Executive and Council, they represent the trainee perspective views on the councils of the surgical Royal Colleges, Intercollegiate Surgical Curriculum Programme, JCST and other committees.

### Global

The Global Anaesthesia, Surgery and Obstetric Collaboration (GASOC) organisation was formed in 2015 following the launch of the Lancet Commission on Global Surgery. This interdisciplinary trainee advocacy group raises awareness about the lack of access to surgical and perioperative care in low and middle-income countries (LMIC) [15]. This has been achieved through online journal clubs, annual conferences and collaborative research projects with active, bilateral participation of LMIC trainees. There are over 1000 members in the UK and globally working on the principles of collaboration, innovation and unity in advancing global surgery agenda.

## Cost of Training

After medical school graduation, trainees are faced with high debts over a 20-year period [16]. The cost of surgical training is high and depends on the surgical speciality one chooses. It is estimated at an average of over £20,000 but costs for Oral and Maxillofacial trainees with dual speciality training can be over £70,000 [17]. Most expenses are related to courses, conferences and exams, with associated travel and attendance fees. The college fees for members and fellows vary between £300 and 500 annually [18, 19]. Membership fees for RCPSG are the lowest at £163 and £408 for fellows [20]. Fees are subsidised for trainees from low- and middle-income countries. The GMC fee for a fully registered doctor with license to practice is around £400 annually [21]. The medical indemnity fees and costs of membership for trade union bodies, like the British Medical Association, will depend on the level of training. With the advancement in technology, the cost of training is likely to rise, and several mitigating factors should be implemented within the training system.

## Technology-Enhanced Surgical Training

The reduction in elective operating as a result of the COVID-19 pandemic has meant that trainees have been forced to look for other opportunities to maintain and develop technical skills [22]. In addition to this, there is growing evidence of the benefits of practicing surgical skills in a simulated setting prior to theatre. In recent times, simulation strategies within surgery have expanded significantly. The introduction of ‘at-home’ laparoscopic simulators, virtual surgical skills courses, augmented and virtual reality (AR and VR) headsets have enabled trainees to develop their motor and cognitive skills in their own time and at their own pace. Many systems are linked to phone or computer-based software that can track their performance and allow either real-time trainer feedback or video uploads. The uptake of robotic surgery is increasing in several hospitals across the UK. However, access to training is limited [17]. The future of surgery report calls for surgical training to change with the ever-evolving advancement in technology [23]. Flexibility in training would allow surgeons to move across different roles and responsibilities.

## Clinical Teaching

At a postgraduate level, surgical teaching can occur through formal departmental programmes or ad hoc case-based learning, in both a clinical and outpatient setting. With the start of the COVID-19 lockdown, face-to-face events were stopped and this provided a significant challenge for trainees to continue their educational activities. Instead, the focus moved to the virtual ‘classroom’, face-to-face presentations moved to online webinars, allowing trainees to attend live and recorded teaching sessions from internationally renowned speakers covering a vast range of topics, from live operating sessions to journal club discussions and examination revision courses. The advantage of an online platform provides flexibility to the modern surgical trainee, enabling them to listen at their convenience and continue the learning process.

Surgical conferences are another key area of postgraduate training that have been transformed into a virtual format as a result of the COVID-19 era. Similar to webinars, online conferences allowed trainees to attend national and international events to present their work at a time when travel was not permitted. New virtual platforms also enabled interaction and networking that face-to-face conferences encourage, providing junior doctors and aspiring trainees the opportunity to ask key questions of their peers and seniors.

## Out of Programme (OOP)

Trainees have several options to take time out of training to learn and develop or take a career break [24]. Out of programme research (OOPR) gives an opportunity to conduct research and gain MD or PhD. Some OOPR can be recognised as part of the training time if recognised by the GMC. Trainees are expected to apply for funding to support the research degree and fieldwork. Trainees can also apply for Out of Programme Training (OOPT) which can be undertaken in the UK and Overseas. Posts recognised by the GMC can be counted towards the training. Out of Programme Experience (OOPE) can be taken in a developing country as defined by the NHS employers [13]. Out of Programme Career break (OOPC) can be undertaken by trainees who need to step out of programme for a reason other than experience, research or training.

## Diversity, Equality, and Inclusion

Surgical training is for everyone. Women in surgery experience various forms of discrimination and a perceived ‘glass ceiling’ during their surgical training [25]. Efforts need to be made to retain women in surgery and tackle inequalities they face [26]. The COVID-19 pandemic has also highlighted the discrimination and racism experienced by the BAME (Black, Asian and Minority Ethnic) trainees [27]. RCS England also called for an independent review on diversity and inclusion and have recommended reforms that need to bring a change [28]. RCS Edinburgh has developed several resources for their #letsremoveit campaign and zero-tolerance approach against bullying and undermining in the workplace [29].

## Wellbeing

Surgical trainees are prone to burnout, stress and psychiatric illness. This has been exacerbated due to COVID-19 effects, leading to poor performance and also impacting on patient care [30]. The Royal Colleges have recognised the negative impact of burnout and offer several online resources, wellbeing webinars and a 24/7 helpline that trainees can access; however, more needs to be done [31, 32]. Training institutions and hospitals continuously need to prioritise the mental health and wellbeing of the surgical trainees. Less than full time (LTFT) training currently exists as an option for reduced working hours. In response to the COVID-19 pandemic, HEE is introducing a new category of LTFT to support mental wellbeing, reduce burnout and improve trainee morale that will be implemented by August 2023 [33].

## Summary

Although there have been many challenges facing surgical trainees, the greatest being the ongoing COVID-19 pandemic, this period has also brought forward new opportunities, accelerated the transition of surgical training into the virtual world and encouraged advancement of novel training solutions, including simulation strategies.
